# Draft Whole-Genome Sequences of 10 Enterotoxigenic *Escherichia coli* Serogroup O6 Strains

**DOI:** 10.1128/genomeA.00564-15

**Published:** 2015-06-04

**Authors:** Vaishnavi Pattabiraman, Cheryl A. Bopp

**Affiliations:** Centers for Disease Control and Prevention, Atlanta, Georgia, USA

## Abstract

Enterotoxigenic *Escherichia coli* (ETEC) is an important cause of diarrhea in children under the age of 5 years and in adults living in developing countries, as well as in travelers to these countries. In this announcement, we release the draft whole-genome sequences of 10 ETEC serogroup O6 strains.

## GENOME ANNOUNCEMENT

Enterotoxigenic *Escherichia coli* (ETEC) is one of the four enteric pathogens that causes more than half of the diarrheal deaths in children under the age of 5 years and adults in developing countries, as well as in travelers to these areas (1, 2). Annually, ETEC is responsible for 200 million diarrheal occurrences and 300,000 to 400,000 deaths, primarily in children under the age of 5 years (3). ETEC strains secrete one or both of the two enterotoxins heat-labile enterotoxin (LT) and heat-stable enterotoxin (ST), which induce water and electrolyte loss in the infected persons, resulting in diarrhea. In addition to these, ETEC strains produce one or more of 25 different colonization factors (CFAs) that mediate the adherence of ETEC to the small intestinal mucosa, leading to diarrhea (4–7). Therefore, the genomic sequences of ETEC strains are of critical importance in studying the evolution of ETEC genomes across different regions of the world, to design and develop vaccines for reducing ETEC-related infant mortality in affected regions, and to evaluate the emerging global ETEC strains in food-borne infections. Previously, we released draft whole-genome sequences of 10 ETEC strains of serogroup O6 (8), and in this announcement, we are releasing the draft whole-genome sequences of 10 additional ETEC strains of O6 serogroup from historical and recent outbreaks (Table 1).

ETEC genomic DNA was extracted from the strains listed in Table 1, subjected to quality control and library preparation, and set up for whole-genome sequencing in MiSeq (Illumina, CA), and raw reads were assembled as indicated in our previous article (8). The sequences were annotated with the NCBI Prokaryotic Genome Annotation Pipeline (http://www.ncbi.nlm.nih.gov/genome/annotation_prok/). The average size of the ETEC genomes in this study was 4.93 Mb, with 4.63 Mb being the smallest genome size (B1020-2; Table 1) and 5.02 Mb being the largest genome size (F6700; Table 1). The average number of coding sequences (CDSs) determined in the ETEC genomes in this study was 4,823 (Table 1). NCBI BLAST tools in CLC Genomics Workbench 7.5.1 identified the classical enterotoxin genes LT and ST1b (Table 1), which were experimentally confirmed by real-time TaqMan PCR (unpublished data) and/or conventional PCR assays for ETEC virulence genes (9).

### Nucleotide sequence accession numbers.

The annotated draft whole-genome sequences of ETEC were deposited in DDBJ/ENA/GenBank, and the accession numbers are listed in Table 1. A detailed report on further analyses of some or all of the draft whole-genome ETEC sequences released (8) to date will be published in the future.

**TABLE 1:** Characteristics of the 10 genomes of ETEC strains^*a*^

ETEC strain	Serotype	NCBI accession no.	No. of contigs	Genome size (bp)	No. of coding sequences	Yr of outbreak	Place of origin
F526	O6:NM	JYHX00000000	371	4,955,905	4,843	1993	USA
F736-c1	O6:NM	JYHY00000000	296	4,954,513	4,835	1993	USA
F6700	O6:H16	JYIA00000000	258	5,027,466	4,953	1999	USA
K1506-c2	O6:H16	JYIB00000000	296	4,939,838	4,826	2004	USA
K1884-sc	O6:H16	JYIC00000000	288	4,993,769	4,871	2005	USA
B144-c1	O6:H16	JYID00000000	335	4,902,430	4,806	1980	USA
2014EL-1181-1	O6:HNT	JYIE00000000	309	4,947,468	4,864	2014	Cruise ship
B1020-2	O6:H16	JYIF00000000	276	4,774,574	4,635	1984	USA
F5524-c2	O6:H16	JYIG00000000	288	4,876,468	4,748	1998	Cruise ship
F6339-c9	O6:H16	JYIH00000000	356	4,908,703	4,857	1998	Cruise ship

a Each strain carried the ETEC virulence genes eltA and st1b.

## References

[1] Okoh AI, Osode AN 2008 Enterotoxigenic *Escherichia coli* (ETEC): a recurring decimal in infants’ and travelers’ diarrhea. Rev Environ Health 23:135–148. doi:10.1515/REVEH.2008.23.2.135. 18763541

[2] Lanata CF, Fischer-Walker CL, Olascoaga AC, Torres CX, Aryee MJ, Black RE, Child Health Epidemiology Reference Group of the World Health Organization, UNICEF 2013 Global causes of diarrheal disease mortality in children <5 years of age: a systematic review. PLoS One 8:e72788. doi:10.1371/journal.pone.0072788. 24023773 PMC3762858

[3] Steffen R, Castelli F, Dieter Nothdurft H, Rombo L, Jane Zuckerman N 2005 Vaccination against enterotoxigenic *Escherichia coli*, a cause of travelers’ diarrhea. J Travel Med 12:102–107. doi:10.2310/7060.2005.12207. 15996455

[4] Dubreuil JD 2012 The whole shebang: the gastrointestinal tract, *Escherichia coli* enterotoxins and secretion. Curr Issues Mol Biol 14:71–82. 22368232

[5] Qadri F, Svennerholm AM, Faruque AS, Sack RB 2005 Enterotoxigenic *Escherichia coli* in developing countries: epidemiology, microbiology, clinical features, treatment, and prevention. Clin Microbiol Rev 18:465–483. doi:10.1128/CMR.18.3.465-483.2005. 16020685 PMC1195967

[6] Gaastra W, Svennerholm AM 1996 Colonization factors of human enterotoxigenic *Escherichia coli* (ETEC). Trends Microbiol 4:444–452. doi:10.1016/0966-842X(96)10068-8. 8950814

[7] Gaastra W, de Graaf FK 1982 Host-specific fimbrial adhesins of noninvasive enterotoxigenic *Escherichia coli* strains. Microbiol Rev 46:129–161. 6126799 10.1128/mr.46.2.129-161.1982 PMC281536

[8] Pattabiraman V, Bopp CA 2014 Draft whole-genome sequences of 10 serogroup O6 enterotoxigenic *Escherichia coli* strains. Genome Announc 2(6):e01274-14. doi:10.1128/genomeA.01274-14. 25523765 PMC4271155

[9] Schultsz C, Pool GJ, van Ketel R, de Wever B, Speelman P, Dankert J 1994 Detection of enterotoxigenic *Escherichia coli* in stool samples by using nonradioactively labeled oligonucleotide DNA probes and PCR. J Clin Microbiol 32:2393–2397. 7814472 10.1128/jcm.32.10.2393-2397.1994 PMC264072

