# Correction for Pattabiraman and Bopp, Draft Whole-Genome Sequences of Nine Enterotoxigenic *Escherichia coli* Serogroup O6 Strains

**DOI:** 10.1128/genomeA.00397-16

**Published:** 2016-05-05

**Authors:** Vaishnavi Pattabiraman, Cheryl A. Bopp

**Affiliations:** Centers for Disease Control and Prevention, Atlanta, Georgia, USA

## AUTHOR CORRECTION

Volume 3, no. 3, e00564-15, 2015. Page 1: The title should read as given above and the number of enterotoxigenic *Escherichia coli* (ETEC) serogroup O6 strains in the study should be given as nine throughout because one of the ETEC strains was confirmed to be a non-O6 strain.

Page 1: The third and fourth sentences of the second paragraph should read as follows. “The average size of the ETEC genomes in this study was 4.92 Mb; B1020-2 (Table 1) had the smallest genome, at 4.77 Mb, and K1884-sc (Table 1) had the largest genome, at 4.99 Mb. The average number of coding sequences (CDSs) in the ETEC genomes in this study was 4,809.”

Page 1: In Table 1, the data for ETEC strain F6700 should be omitted because F6700 is a non-O6 strain.

